# The role of gut microbiota in modulating immune responses in chronic liver disease: a systematic review and meta-analysis

**DOI:** 10.3389/fimmu.2025.1556576

**Published:** 2025-05-16

**Authors:** Eyad Gadour, Khalid Jebril Shrwani, Zeinab Hassan, Bogdan Miutescu

**Affiliations:** ^1^ Multiorgan Transplant Centre of Excellence, Liver Transplantation Department, King Fahad Specialist Hospital, Dammam, Saudi Arabia; ^2^ Internal Medicine Department, School of Medicine, Zamzam University College, Khartoum, Sudan; ^3^ Department of Medical Virology, Public Health Authority, Southern Sector, Jazan, Saudi Arabia; ^4^ Department of Internal Medicine, Stockport Hospital National Health Sevices (NHS) Foundation Trust, Manchester, United Kingdom; ^5^ Department of Gastroenterology and Hepatology, Victor Babes University of Medicine and Pharmacy, Timisoara, Romania; ^6^ Advanced Regional Research Centre in Gastroenterology and Hepatology, Victor Babes University of Medicine and Pharmacy, Timisoara, Romania

**Keywords:** gut microbiota, immunity; immune responses, chronic liver diseases, TNF, liver cirrhos

## Abstract

**Introduction:**

The gut microbiota plays a crucial role in regulating immune responses and maintaining a balance within the gut-liver axis. In patients with chronic liver disease (CLD), alterations in gut microbiota have been linked to disease progression and impaired immune function. This study aimed to evaluate the impact of gut-modulating therapies on the immune responses of patients with CLD.

**Method:**

Two independent authors conducted a comprehensive literature search using complementary strategies to identify relevant articles published until March 2025. Review Manager Software (RevMan 5.4) was used for data analysis, and the results were presented using forest plots.

**Results:**

Of the 373 identified studies, 16 were included in the analysis. The findings revealed that gut microbiota-modulating therapies significantly reduced tumor necrosis factor-α (TNF-α) levels compared to control interventions (standardized mean difference [SMD], -0.60; 95% confidence interval [CI] [-0.93, -0.23] p = 0.001), with similar results observed at the 6-month follow-up (SMD -1.3; 95% CI [-2.1, -0.4] p = 0.004). Interleukin-6 (IL-6) levels showed no significant change between the groups (SMD, -0.67; 95% CI [-1.5, 0.12) p = 0.09). C-reactive protein (CRP) levels were significantly reduced by gut-modulating therapies (SMD -1.057; 95% CI [-1.493, -0.621] p = 0.0005), with consistent results at 1- and 6-month follow-up. Changes in interferon-gamma (IFN-γ) and IL-18 levels and cellular immunity were also assessed.

**Conclusion:**

This study highlights the importance of gut microbiota in modulating immune responses in patients with CLD and demonstrates the effectiveness of long-term gut-modulating therapies in reducing inflammatory markers. While CRP and TNF-α levels decreased, changes in IL-6 levels were inconsistent, warranting further research to elucidate the impact of gut microbiota-modulating therapies on this biomarker.

## 1 Introduction

Chronic liver disease (CLD) is defined by a progressive decline in liver function persisting for more than six months, with cirrhosis representing its terminal stage. The principal etiologies of CLD encompass alcoholic liver disease (ALD), non-alcoholic fatty liver disease (NAFLD), chronic viral hepatitis, genetic predispositions, autoimmune disorders, and certain pharmacological agents (1). CLD is among the leading causes of mortality worldwide, accounting for 2.2% of deaths, with 1.32 million fatalities reported in 2017 (2). While alcohol consumption is commonly linked to CLD in numerous developed countries, the hepatitis B virus (HBV) is the predominant cause in sub-Saharan Africa and Asia, and NAFLD is becoming increasingly prevalent globally (3). CLD is characterized by persistent inflammation, which plays a critical role in the disease’s progression and associated complications. The inflammatory response in CLD is intricately linked to an imbalance in gut microbiota, resulting in a continuous cycle of hepatic injury and immune system dysfunction. This relationship is particularly evident in the gut-liver axis, where alterations in gut permeability and bacterial translocation contribute to hepatic inflammation.

Gut microbiota plays an important role in the human body because it is linked to the overall good health of an individual, essentially maintaining the structural integrity of the gut and immune regulation, with the main types, including Bacteroidetes and Firmicutes (4). Disrupted gut bacterial composition has been associated with the occurrence of various inflammatory conditions, including CLD (5). Intestinal barrier integrity is essential, as exposure of the immune system to the gut microbiota causes disease and inflammation (5). The interaction is achieved through the portal vein, connecting the liver and the intestines, creating a network (gut–liver axis) of exchange of bile and intestinal products, such as nutrients (6).

Research has increasingly associated gut microbiota with modulating the immune response in CLD, as these bacterial components signal the toll-like receptors (TLRs), activating inflammation. TLR overstimulation leads to tolerance, inhibiting immunity, persistent inflammation, and potentiating CLD (7). Changes in interferon-gamma (IFN-γ) levels have been observed in CLD patients, with studies showing altered production of this cytokine in response to gut microbiota dysbiosis. Similarly, changes in IL-18 levels have been reported in CLD, reflecting the complex interplay between the gut microbiome and the immune system. Furthermore, changes in cellular immunity, including alterations in the number and function of various immune cell populations such as T cells and natural killer cells, have been documented in CLD patients.

Additionally, patients with NAFLD have less microbiota, fewer CD4 and CD8+ lymphocytes, and higher TNF and IFN expression than the healthy cohort. In chronic HBV, intestinal integrity and bacterial composition alteration are the genesis of systemic immune activation TLR activation due to systemic endotoxin presence, catalyzing the inflammatory cascade, leading to CLD (8). Furthermore, patients with HBV-CLD have a decreased number of beneficial bacteria and an increased number of bacteria associated with inflammation (9). Subsequently, changes in peripheral blood mononuclear cells (PBMCs) were observed after interaction with HBV-CLD, revealing that the microbiome and metabolome showed marked alterations in the gut bacteria in HBV-CLD caused by disease progression (10).

Currently, treatment approaches target different causes of CLD. CLD has a wide range of treatment regimens, with probiotics and symbiotic therapy improving alanine aminotransferase (ALT) levels, reducing the immune response through inflammation (11). In another study, using Bifidobacterium with FoS plus lifestyle modification decreased ALT levels and NASH activity (12). Mofidi et al. also concluded that treatment supplementation of patients with NAFLD with symbiotics improved hepatic function (13). Moreover, research has also led to the identification of yogurt probiotics in managing NAFLD, as its supplementation led to a reversal of minimal hepatic encephalopathy (MHE) and increased adherence. Subsequently, Bajaj et al. reported that the simultaneous administration of symbiotics and vitamin E led to good outcomes in patients with NAFLD (14).

Through our diverse approach, this study aimed to analyze the alterations in gut microbiota composition in CLD and their relationship to the immune response by summarizing the evidence of changes in the immune response as a result of therapies that aim to restore the normal gut microbiota composition.

## 2 Methodology

### 2.1 Protocol and registration

This systematic review and meta-analysis was conducted in accordance with the Preferred Reporting Items for Systematic Reviews and Meta-Analyses (PRISMA) 2020 guidelines. However, the study protocol was not registered in any publicly accessible database.

### 2.2 Literature search

Two independent researchers conducted a literature review employing two complementary methodologies to identify all articles published up to March 2025. Initially, a comprehensive electronic search was executed using predefined criteria across three databases: Google Scholar, Science Direct, and PubMed. This search utilized the Boolean operators “AND” and “OR” to effectively combine keywords. The complete search strategy for PubMed included: (Gut microbiota OR Gut microbiome) AND (Probiotics OR Synbiotics OR Antibiotics) AND (Chronic liver disease OR Alcoholic hepatitis OR Viral hepatitis OR Cirrhosis OR Alcoholic steatohepatitis OR Non-alcoholic fatty liver disease) AND (Immune response OR Inflammatory markers OR Inflammatory cytokines OR neutrophil function). Furthermore, the reviewers manually examined the reference lists of selected articles to identify any studies that might have been overlooked, thereby ensuring comprehensive coverage of relevant literature.

### 2.3 Eligibility criteria

After retrieving all the articles from the three databases, they were assessed independently based on the predefined eligibility criteria. A study was included if it meets the following inclusion criteria:

1. Studies published in English.
2. Studies including patients with CLD (including those with cirrhosis, NAFLD, ALD, and chronic hepatitis).
3. Studies investigating the efficacy of different gut-modulating therapies, such as symbiotics, antibiotics, and probiotics.
4. Studies with a comparator, including placebos or other control interventions.
5. Studies designed as either randomized controlled trials (RCTs), observation cohort studies, or case-control studies.
6. Studies reporting changes in immune function, e.g., through changes in inflammatory cytokines or neutrophil activities.

We excluded the studies meeting the following exclusion criteria:

1. Studies not investigating any of the gut microbiota-altering therapies.
2. Studies without any comparator arm to the interventional group.
3. Studies not reporting any of the outcomes of the immune response after gut microbiota modulation.
4. Other secondary studies, case reports, and letters to the editor.

### 2.4 Study selection and data extraction

Independent reviewers conducted the study selection through a multi-stage process, which involved the removal of duplicate articles, the evaluation of titles and abstracts, and the examination of full texts. Initially, the abstracts of the articles remaining after the elimination of duplicates were assessed against the inclusion criteria. Articles that satisfied these criteria were included, while those with uncertain eligibility underwent a full-text review. Following the selection process, the reviewers independently extracted relevant data from the included studies using extraction forms that had been pilot-tested. Data were collected for all time points and utilized in the analysis, including the first author’s last name and publication year, study setting, design, study inclusion criteria, sample size, average ages, and reported outcomes.

### 2.5 Statistical analysis

We used the statistical software Review manager (RevMan 5.4) for the meta-analysis, The following outcomes were assessed: changes in the serum levels of interleukin-6 (IL-6), C-reactive proteins (CRPs), IL-18, interferon-γ, and tumor necrosis factor-α (TNF-α), which were analyzed using the standardized mean difference (SMD). Subsequently, the results were presented using forest plots. Statistical significance was determined at p ≤ 0.05. Due to the expected high heterogeneity of the outcomes, we used the random-effects model for the analysis. I^2^ statistics was used to assess heterogeneity.

### 2.6 Risk of bias assessment

ROB 2 tool (Cochrane Collaboration) was used to assess the risk of bias (ROB). Using this tool, two authors independently analyzed the ROB in each of the studies. They analyzed the bias that may arise from each of the five key domains, including randomization of the study participants, blinding of participants and investigators, reporting of the study outcomes, and missing data. Using the five domains, the authors assigned the overall ROB for each of the domains as either “high,” “low,” or “some concerns” based on various factors. In case of any disagreement between the reviewers on the ROB in a particular domain, a consensus was reached through the intervention of a third reviewer not involved in the ROB appraisal method. The Newcastle–Ottawa scale was utilized to analyze the methodological quality of observational studies.

## 3 Results

### 3.1 Search results

**Figure 1:**
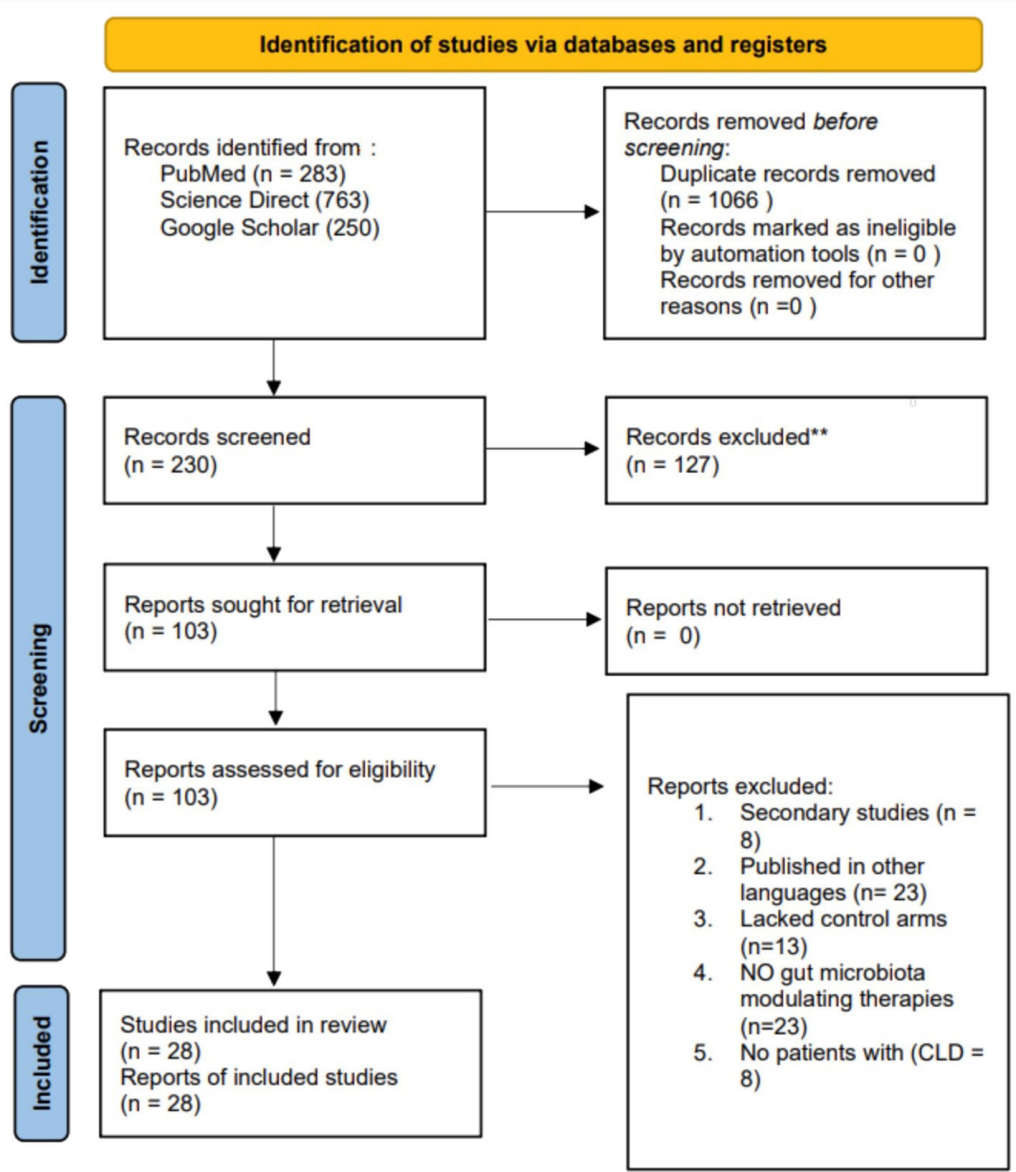
A PRISMA flow diagram summarising the search strategy.

An extensive literature search yielded 1,296 articles from the databases. Following the assessment for duplicates, 1,066 redundant articles were eliminated. Subsequently, 230 abstracts were reviewed for their relevance to the study topic, resulting in the exclusion of 127 studies. The remaining 103 articles were retrieved and evaluated based on predetermined eligibility criteria, of which only 28 met the inclusion criteria and were incorporated into the review. The remaining 75 articles were excluded for the following reasons: 23 were not published in English, 13 lacked control arms, 8 were secondary studies such as reviews, 23 did not include microbiota-modulating therapies as one of their interventions, and 8 excluded patients with CLD. **Figure 1** presents a PRISMA diagram that summarizes the search strategy.

### 3.2 Characteristics of the included studies

This study includes 28 studies conducted in different countries, including the United Kingdom, Austria, Malaysia, Iran, the United States of America, Japan, Spain, Italy, India, and Ukraine. Among the included studies, 27 were RCTs (12–38), whereas one was an observational study (11). The various interventions investigated across the studies included probiotics and symbiotics. **Table 1** shows the characteristics of the included studies.

**Table 1:** Characteristics of the included studies.

Author ID	Study setting	Study design	Inclusion criteria	Intervention	Characteristic of interventions	No of participants	Mean ages	Immune marker analyzed
Horvath et al., 2016 (16)	Austria	Randomized clinical trial	Patients aged 18–80 from years in the outpatient department with cirrhosis of any etiology	Probiotic or Placebo	6g of multispecies probiotic or placebo daily for the first 6 months	92	49	Increased serum neopterin levels and production of ROS, Phagocytic capacity of monocytes increased after 3 months, low infection rate
Mohamad Nor et al., 2021 (17)	Malaysia	RCT	Patients aged ≥18 years with ultrasound diagnosis of fatty liver, ALT of >35 and 25 IU/L for men and women, respectively.	Probiotic or placebo	1 sachet daily for 6 months	32	Above 18	Reduction in expression of CD4+ T lymphocytes and ZO-1 In placebo group
Roman et al., 2019 (19)	Spain	RCT	Patients from the outpatient department who had cirrhosis and cognitive dysfunction and fell	Probiotic or placebo	4.4g sachet twice a day for 12 weeks	121	NR	Decrease in CRP and TNF- alpha, Neutrophil Oxidative Burst increased after PMA stimulation, decrease in biomarkers serum FABP-6 and urinary Claudian-3 in the probiotic group.
Aller et al., 2011 (25)	Spain	RCT	Patients with NAFLD diagnosed with liver disease through liver biopsy	Probiotic tablet- *Lactobacillus bulgaricus* and *Streptococcus thermophilus*.	Probiotic tablet- *Lactobacillus bulgaricus* and *Streptococcus thermophilus*.		14–no differences in age and gender distribution	
					Placebo		14–both males and females	
Bajaj et al., 2008 (14)	United States of America (USA)	RCT	Non-alcoholic patients with minimal hepatic encephalopathy (MHE)	Probiotic yogurt: 17 patients	12 ounces per day for 60 days	25	NR	IL-6 and TNF-alpha levels at baseline and end had no change
Kobyliak et al., 2018 (20)	Ukraine	RCT	Patients with NAFLD aged between 18 and 65 years with a BMI of ≥25 kg/m^2^, diagnosed based on clinical examination, laboratory values of lipid and carbohydrate metabolism, ALT/AST ratio, and ultrasound	Mult Probiotic symbitter	1 sachet (10g) of probiotics every day for 8 weeks.	58	42	TNF-alpha, IL-1 Beta, IL-6, IL-8, IFN- gamma.
				Placebo		28		
Dhiman et al., 2014 (24)	India	RCT	Patients with cirrhosis who had recovered from an episode of hepatic encephalopathy (HE) during the past month.	Probiotic VSL#3 – 66	1 sachet daily for 6 months	130	NR	Fasting blood ammonia levels and plasma indole, TNF-alpha, IL-1Beta, IL-6, plasma renin, aldosterone, BNP levels decreased
				Placebo–64	1 corn flour sachet for 6 months			Fasting blood ammonia and aldosterone levels increased.
Bajaj et al., 2014 (21)	United States of America (USA)	RCT	Patients with cirrhosis and MHE aged between 18 and 65 years with histological evidence with radiology and endoscopy whose disease had been stable for 6 months with no treatment changes.	Probiotic *Lactobacillus* GG (LGG) – 14	1g LGG of >50 million CFU and followed up for 8 weeks	30		Reduced TNF-alpha and endotoxemia in stool
				Placebo				
Ekhlasi et al., 2017 (23)	Iran	RCT	Patients aged between 25 and 64 years with NAFLD in both males and females with a body mass index (BMI) between 25 and 35 kg/m^2^	Symbiotic–15	2 capsules containing 1g per day after meals for 8 weeks	60	44.5	Decrease in serum AST
				Symbiotic + alpha-tocopherol-like placebo + alpha-tocopherol – 15	400 IU daily for 8 weeks			Decreased AST
				Alpha-tocopherol + symbiotic-like placebo–15				Reduced TNF-alpha
Eslamparast et al., 2014 (22)	Iran	RCT	Patients with NAFLD whose diagnosis was based on ultrasound examination, steatosis presence, and persistent elevation of alanine aminotransferase (ALT) >60 U/L 6 months before the study.	Symbiotic Placebo	Synbiotic or identical placebo capsule twice a day for 28 weeks.	52		The symbiotic group had lower ALT, AST, and GGT levels than the placebo group, and both groups had decreased BMI and WHR.
Macnaughtan et al., 2020 (15)	United Kingdom (UK)	RCT	1.) Patients between the ages of 18 and 78 years who were abstinent from alcohol 2 weeks before screening and had alcoholic cirrhosis	*Lactobacillus casei* Shirota	65ml of LcS, 3 times a day for 6 months	46	57	Significant involvement in the production of Phorbol 12-myristate 13-acetate (PMA)- induced neutrophil ROS
				Placebo	65ml of placebo, 3 times a day for 6 months			
Stadlbauer et al., 2008 (11)	UK	Observational cohort study.	*Lactobacillus casei* Shirota (LcS)	Stable outpatient department patients with alcoholic cirrhosis	65 ml each 3 times a day	12	NR	Phagocytic capacity increases after 28 days of treatment, and IL-10, STNFR1, and STNFR2 levels are elevated. TLR 2 was elevated
			Patients with cirrhosis not on LcS		NR	8		Normal phagocytic capacity at the start and after 28 days
			Healthy cohorts		NR	13		Normal phagocytic capacity throughout the study.
Malaguarnera et al., 2011 (12)	Italy	RCT	If a patient had sonography results suggesting hepatic steatosis abdominal serum transferase. Patients with a liver biopsy consistent with non-alcoholic steatohepatitis (NASH). Patients not on metformin, vitamin E, or thiazolidinedione	Bifidobacterium longum + lifestyle changes + Fructo-oligosaccharides (FOS) or Placebo + Lifestyle modification	2.5g Bifidobacterium longum and FOS (or placebo), vit B1 (1.4mg), vit B2 (1.6mg), vit B6 (2.0mg) for 24 weeks.	34	NR	Baseline decrease in AST, ALT, LDL. Elevated HDL compared to placebo. Reduced TNF-alpha, CRP, serum AST, HOMA-IR, and serum endotoxin.

Koga et al., 2012 (18)	Japan	RCT	Patients with alcoholic liver cirrhosis.	Y400 served twice a day during the first half of the 4 weeks Placebo	Y400 twice a day during the first half of the four weeks or Placebo twice a day during the first half of four weeks.	37	NR	Transthyretin increased, obligate anaerobic bacteria increased, transferrin and albumin, although insignificant, increased, and CRP decreased. In the placebo group, h-CRP and IL-6 increased insignificant
Ayob et al., 2023 (26).	Malaysia	RCT	Patients aged 18 years and above with ultrasound diagnosis of fatty liver, with baseline-controlled attenuated ALT of more than 35 IU/L for males and 25IU/L for females, who had chronic liver disease.	Probiotic or Placebo	1 sachet twice a day for 6 months.	24	NR	The probiotic group had an increase in Actinobcterium phylum months and a decrease in microbiota. Decrease in IFN-gamma, an increase in TNF-alpha and IL-6, and a decrease in P in O-1 protein.
						24		The placebo group had a decrease in the Phyluhdm, increased microbiota Decrease in IFN-gamma., no difference in T, deF-alpha, decrease in ZO-1 protein.
Mofidi et al., 2017 (13)	Iran	RCT	Patients aged ≥18 years with NAFLD, with no history of alcohol consumption, no signs of other acute or chronic liver diseases	Maltodextrin or Placebo	1 capsule twice a day for 28 weeks	50	NR	TNF-alpha increased, decreased ALT, JNK, NF-kB, fatty acid oxidation
				Symbiotic supplementation	NR			
Escouto et al., 2023 (27).	Brazil		Patients with NASH of above 18 years with a BMI of 32.7 kg/m squared	Probiotic Placebo	1 capsule daily for 6 months	48	58	APRI score decreased in the Probiotic group, decreased AST, ALT concentration
Roussel et al,. 2022 (28).	France	RCT	Patients with resectable hepatocellular carcinoma	Probiotic Placebo	1 oral probiotic sachet tablet twice daily	64	NR	TNF-alpha levels increased in the probiotic group, and IL-b Levels increased.
Mitrovic et al., 2024 (29).	USA	RCT	MASLD patients with elastomeric attenuation coefficient greater than 0.63 Db/cm with ALT above 40U/L for men and 35U/L for women	Synbiotic Placebo	6.4g inulin and lactobacillus daily for 12 weeks	84	NR	Increase in CRP in the symbiotic group,
Laghi et al. (30),.	Italy	RCT	Patients aged 18 years and above in the outpatient department with cirrhosis	Probiotic	BCAA 10g 30 min before exercise, one sachet of probiotic every 12 hours, or control for 12 months.	37	NR	Decrease in dimethyl sulfone and increase in malonate, ornithine, and valine.
Manzhalii et al., 2022 (32).	Ukraine	RCT	Patients aged 18 to 65 years with cirrhosis and had two or more episodes of HE in the last 6 months.	Probiotic	Probiotics for first 4 days, one capsule 4 times a day, then twice a day for 1 month	15	NR	Probiotic EcN and rifaximin had reduced proinflammatory cytokines INF-gamma, IL-6, and IL-8.
				Lactulose	30–60 mL in 2 or 3 divided doses.	15		
				Rifaximin	500mg twice per day.	15		
Zhang et al., 2025 (31).	China	RCT	Patients aged 18–65 years with a long-term history of alcohol intake of more than 1 year,	Probiotic Placebo	BC99 3G per day, or placebo 3g daily for 60 days.	72	NR	ALT, AST, glutamyl transpeptidase, serum bilirubin, blood urea nitrogen, uric acid, TNF-alpha, and IL-6 with an increase in IL-10 in the probiotic group
Sepideh et al., 2015 (33).	Iran	RCT	NAFLD patients between 18–65 years	Probiotic Placebo	2 capsules of probiotic or placebo every day for 8 weeks	50	NR	Insulin, insulin resistance, TNF-alpha, and IL-6 were decreased
Jayakumar et al., 2013 (35).	Canada	RCT	Patients 18 years and above with a diagnosis of cirrhosis were diagnosed using radiology or histology.	Probiotic or Placebo	2 sachets of either VSL#3 or Placebo for 8 weeks	17	NR	Changes in IL-6, IL-8, and IL-10.
Abhari et al., 2020 (34).	Iran	Randomized double-blind control trial	Patients aged 18–75 years old with steatosis on fibro scan and high concentration of ALT >1.5 times upper limit, no history of alcohol consumption, diabetes, hepatitis, weight loss, or bariatric surgery.	Synbiotic or placebo	1 capsule of symbiotic plus 0.4g insulin per day	26	NR	ALT and glutamine transaminase decreased, and TNF-alpha and factor-kB activity was reduced.
Gupta et al., 2013 (36).	India	Randomized double-blind placebo-controlled trial	Patients with large esophageal varices diagnosed by clinical, biochemical, and radiological criteria with or without liver biopsy	VSL#3 + propanol	900 billion CFU daily	94	NR	Decrease in TNF-alpha, IL-6 and NO showed decrease in concentration.
				Placebo + propanol	1 sachet containing corn starch			
				Norfloxacin caps (9) + propanol	200mg			
Patel et al., 2022 (37).	UK	Placebo-controlled double-blind study	Patients of ages 18–75 with cirrhosis and chronic HE	Rifaximin-α	550 mg twice a day for 90 days Placebo for 90 days	19	NR	Reduced neutrophil TLR-4 and TNF-alpha. TNF-alpha and interleukin-17- enriched intestinal microenvironment
				Placebo		19		
Kimer et al., 2022 (38).	Denmark	RCT	Patients 18 years and above with alcohol intake of more than three units per day for more than 3 months or more than 10 units per day for more than 1-month, rapid jaundice development, bilirubin >50 μmol/liter	Standardized medical therapy (SMT)	400mg three times a day for 2–4 weeks	32	NR	IL-6, IL-8, IL-10, and TNF-alpha were elevated but decreased over time. Inflammatory markers and amino acids were high in the early stages
				SMT + rifaximin	550 mg three times a day for 4 weeks.			

NR, not reported.

### 3.3 Methodological quality and ROB of the included studies

**Figure 2:**
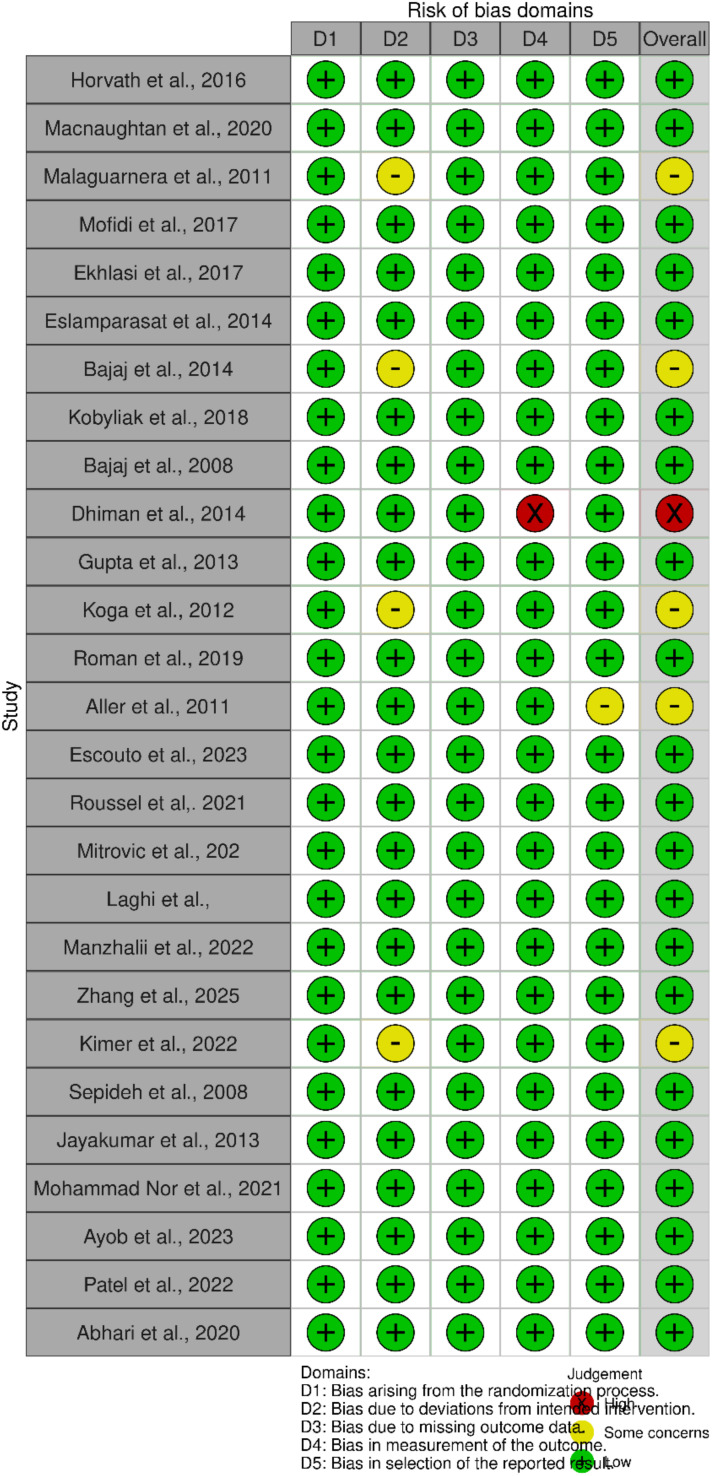
A risk of bias summary showing the risk of bias of the various studies.

The methodological quality of the included observational study was fair (**Table 1**). Most included studies showed low ROB (**Figure 2**). A study by Dhiman et al. (24) had high ROB contributed by the high ROB under outcome measurement (**Table 2**).

**Table 2:** A NOS summarizing the methodological quality of the selected observational study.

Study ID	Selection	Comparability	Reporting	AHRQ standard
Stadlbauer et al., 2008 (11)	3	1	2	Fair

### 3.4 TNF-α levels

**Figure 3:**
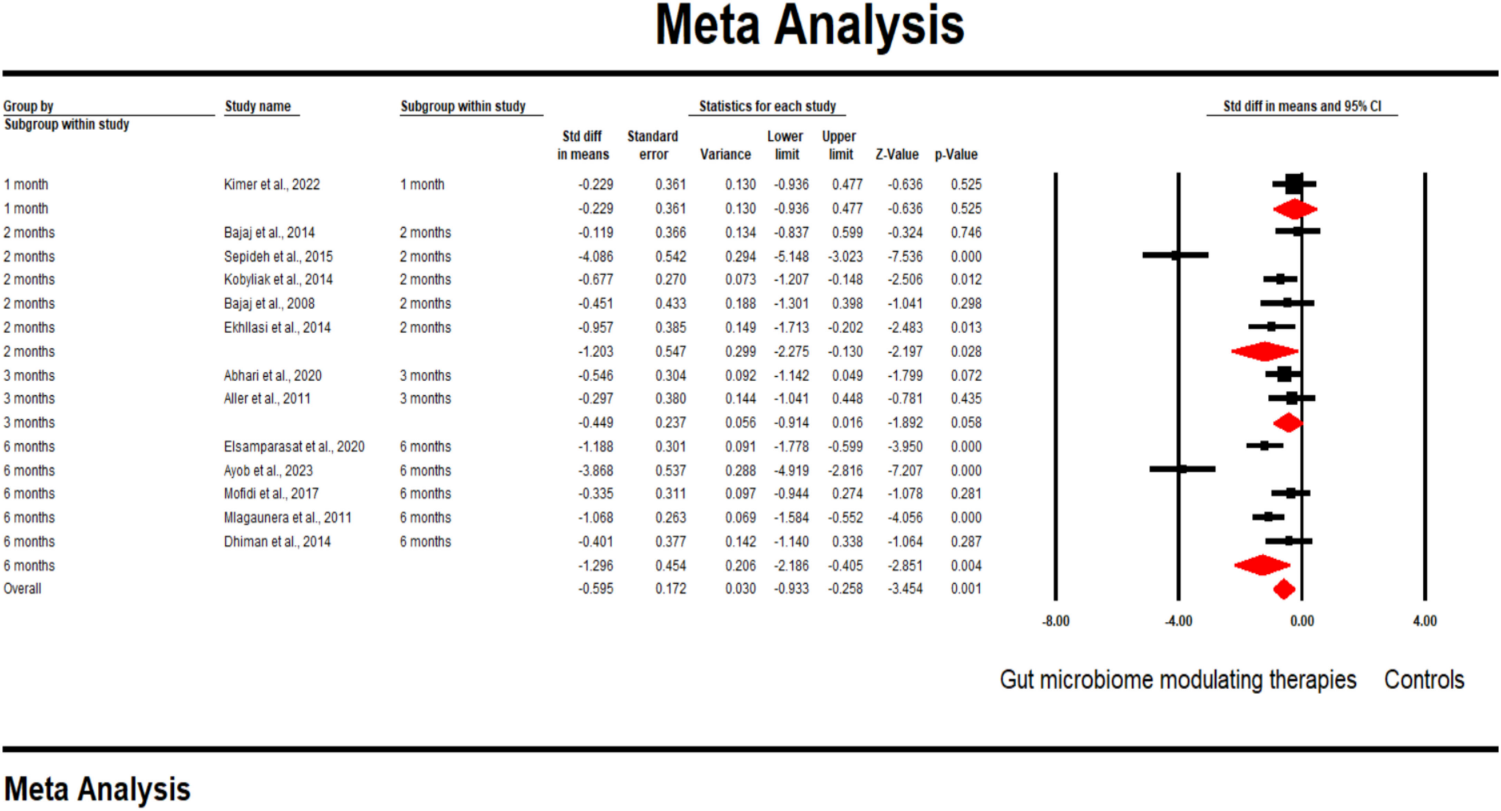
A forest plot showing changes in Changes in TNF-α levels.

13 studies reported the outcome of changes in TNF-alpha levels. A pooled analysis of the outcomes showed that gut microbiota-modulating therapies significantly decreased the TNF-α levels compared to placebo (SMD -0.60; 95% CI [-0.93, -0.23] p = 0.001). A subgroup analysis of the outcome found similar results during the 2- months follow-up (SMD -1.2; 95% CI [-2.3, 0.13] p = 0.03) and 6-month follow-up periods (SMD -1.3; 95% CI [-2.1, -0.4] p = 0.004). However, no significant difference was observed in the 1-month and 3-month follow-up period (SMD -0.23; 95% CI [-0.94, 0.48] p = 0.53) and (SMD -0.45; 95% CI [-0.91, 0.02] p = 0.06), respectively (**Figure 3**).

### 3.5 Changes in 1L-6 levels

**Figure 4:**
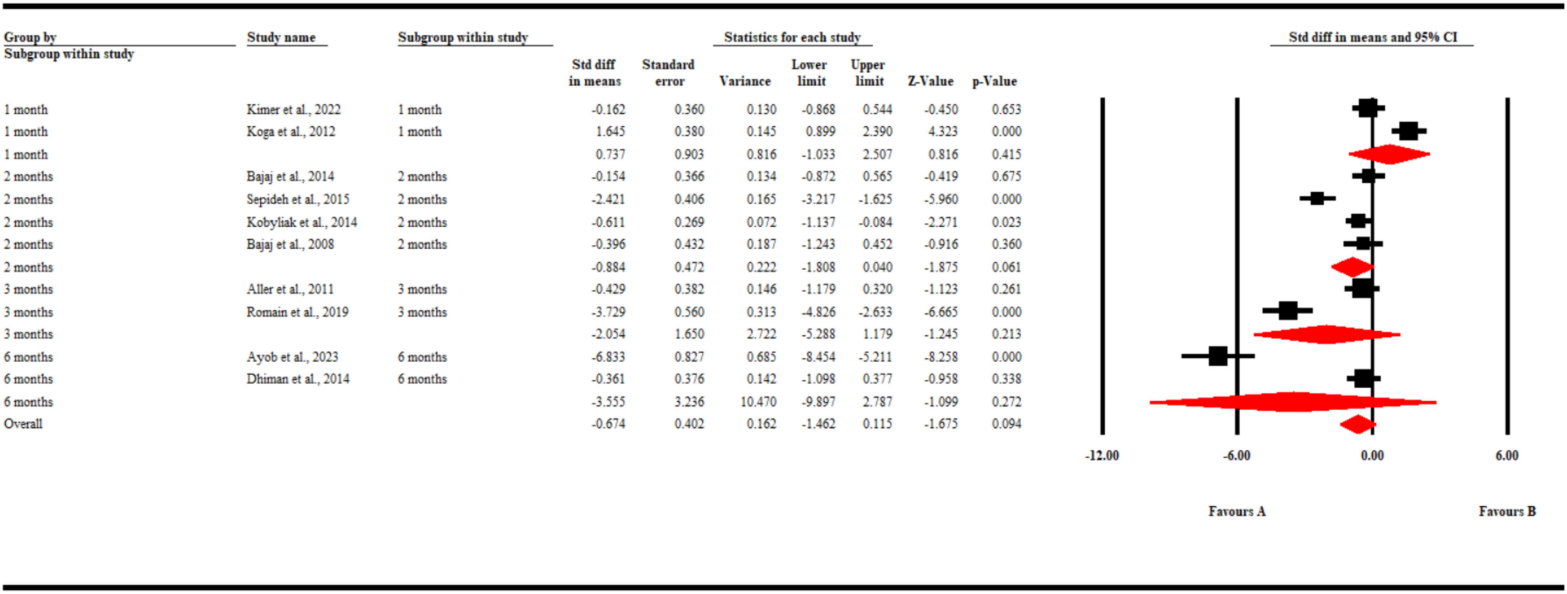
A forest plot showing changes in IL-6 levels.

Nine studies reported changes in IL-6 levels. Our pooled analysis found no significant difference in the changes in IL-6 levels in both groups of patients (SMD -0.67; 95% CI [-1.5, 0.12) p = 0.09). Similarly, a subgroup analysis found that during the different time points in follow-up, gut microbiota-modulating therapies failed to significantly decrease the levels of Il-6 compared to placebo (SMD0.74; 95% CI [-1.03, 2.51] p = 0.42) at 1 month, (SMD -0.88; 95% CI [-1.81, 0.04] p = 0.06) at 2 months, (SMD -2.05; 95% CI [-5.29, 1.18] p = 0.21) at 3 months, and (SMD -3.56; 95% CI [-9.9, 2.79] p = 0.27) at 6 months (**Figure 4**).

### 3.6 CRP levels

**Figure 5:**
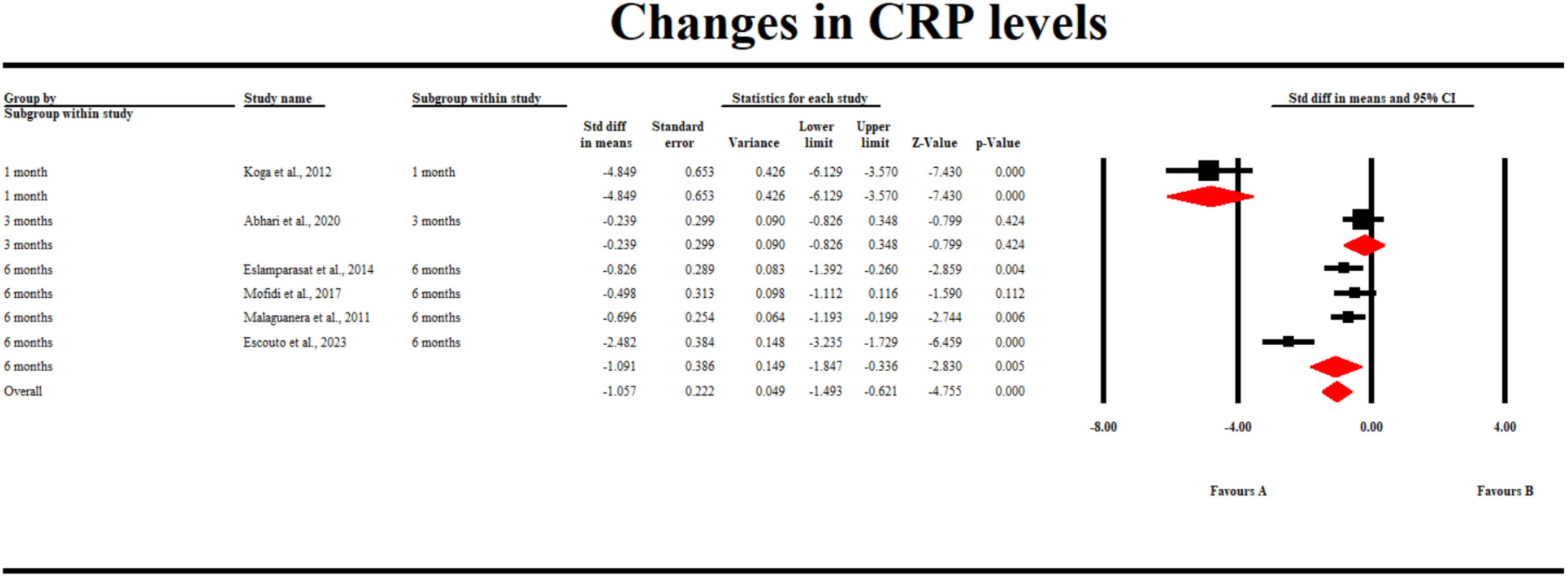
A forest plot showing changes in CRP levels.

Only 6 studies reported changes in CRP levels. Our pooled analysis found that gut microbiota-modulating therapies significantly reduced serum CRP levels compared to placebo (SMD -1.057; 95% CI [-1.493, -0.621] p = 0.0005). Furthermore, similar results were observed at the 1-month and 6-month follow-up periods (SMD -4.85; 95% CI [-6.13, -3.57] p < 0.001) and (SMD -1.01; 95% CI [-1.85, -0.34] p = 0.005). However, no significant difference was observed after 3 months of interventions (SMD -0.239; 95% CI [-0.83, 0.35] p = 0.424) (**Figure 5**).

### 3.7 Changes in interferon-gamma (IFN-γ) levels

**Figure 6:**
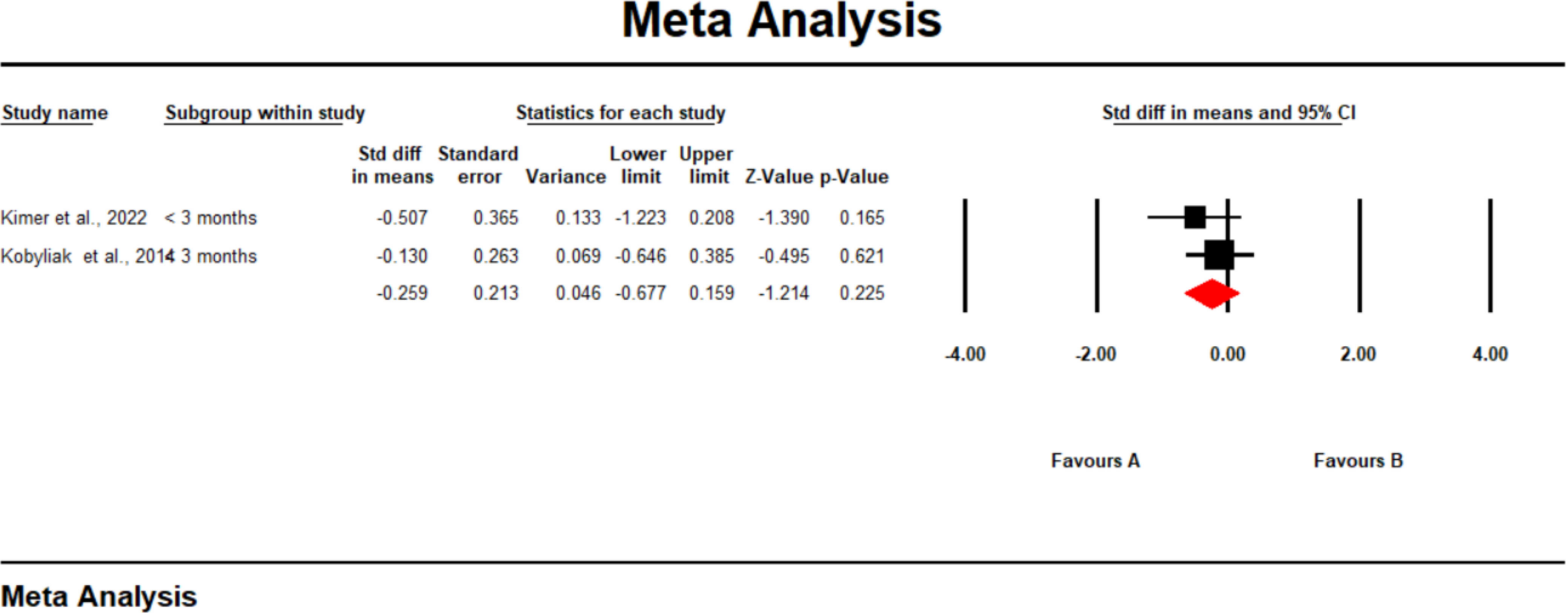
A forest plot showing changes in Interferon-gamma.

Two studies reported changes in interferon-γ. Our analysis found that gut-modulating therapies did not significantly affect the levels of IFN-γ in patients with chronic liver disease (SMD -0.259; 95% CI [-0.677, 0.159], p = 0.225) (**Figure 6**). Despite some variability across individual studies, the pooled results suggest that the interventions had no statistically significant impact on IFN-γ expression. This indicates that while gut microbiota-targeted treatments may influence other inflammatory markers, their effect on IFN-γ remains inconclusive and warrants further investigation through larger, well-designed clinical trials.

### 3.8 Changes in IL-18 levels

**Figure 7:**
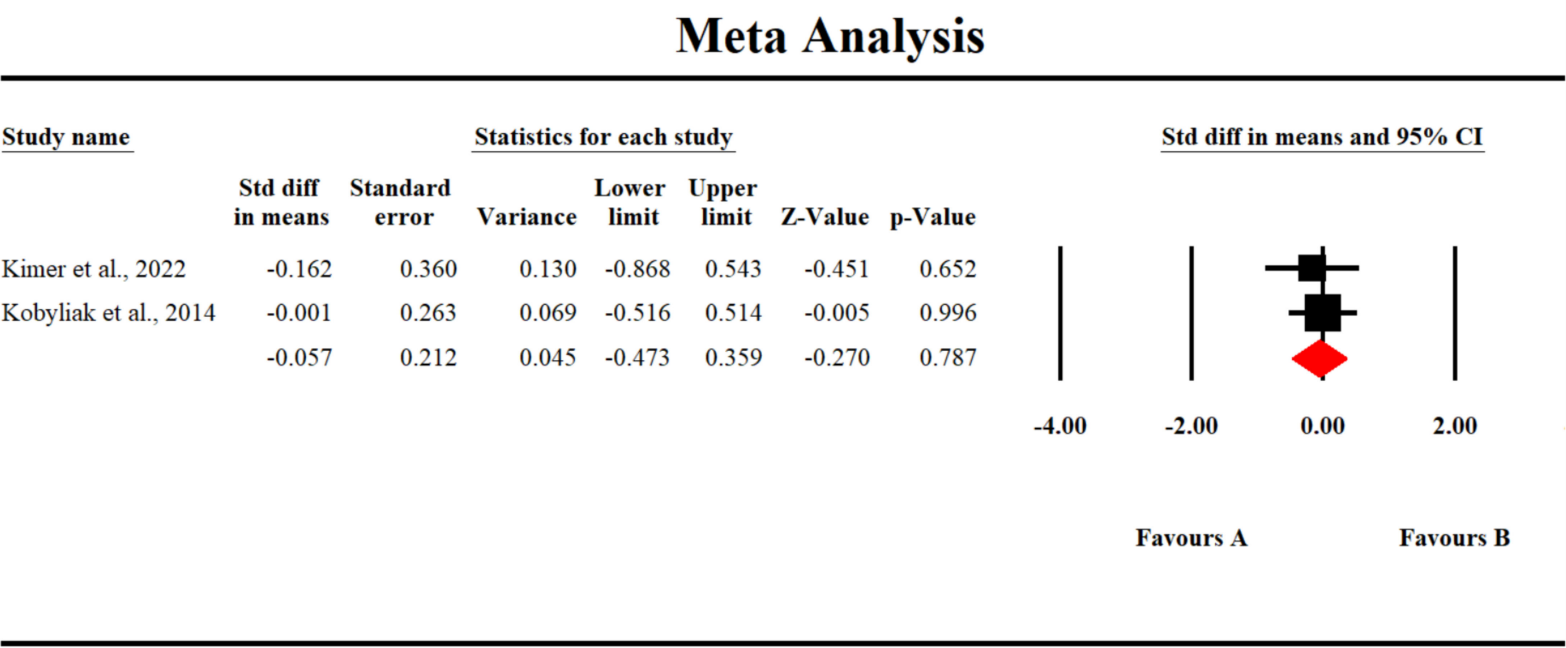
Changes in Il-8 levels.

Two studies reported changes in IL-18 levels. Our pooled analysis showed that gut microbiome-modulating therapies did not significantly affect the levels of IL-18 in patients with chronic liver diseases (SMD -0.06; 95% CI [-0.47, 0.36] p = 079) (**Figure 7**).Our pooled analysis revealed that gut microbiome-modulating therapies did not significantly alter IL-18 levels in patients with chronic liver diseases (SMD -0.06; 95% CI [-0.47, 0.36] p = 0.79) (**Figure 7**). This finding was based on two studies that reported changes in IL-18 levels. The lack of significant effect suggests that these therapies may not have a substantial impact on this particular inflammatory marker in the context of chronic liver diseases.

### 3.9 Changes in cellular immunity

Due to differences in the reporting of cellular immunity changes, we could only do a narrative synthesis of the reported outcomes. Three of the included studies reported on the changes in immunohistochemistry of CLD patients. Romain et al. found that the neutrophil oxidative burst significantly increased in CLD patients treated with probiotics after stimulation with PMA (19). Similar results were observed by Horvath et al. However, they noted that the resting oxidative burs had no significant changes. Horvath et al. also found that the phagocytic activity of the neutrophils in the overall study population decreased with time in all the patients. This was, however, not observed in the phagocytic capacity of the monocytes, which increased after 3 months in the probiotic group (16). Lastly, Nor et al. found that the expression of CD4+ lymphocytes did not change in both groups but observed a decrease in the levels of CD8+ lymphocytes in patients treated with placebo (17).

## 4 Discussion

Modulation of the gut microbiota is crucial to maintain the gut–liver axis, as it prevents the interaction of the gut microbiomes with inflammatory cells, preventing CLD. The most frequent cytokines include TNF-α, IL-6, and CRP. Therapeutic intervention in patients with CLD aims to decrease the levels of these pro-inflammatory cytokines. Therefore, treatment of patients with CLD includes diet, probiotics, or fecal microbiota to enhance normal gut bacterial growth, may relieve gut dysbiosis, and improve the prognosis of patients with CLD (39).

TNF-α is a pro-inflammatory cytokine causing the immunopathogenesis of various diseases and organs, such as the liver, where it is involved in liver inflammation and apoptosis of hepatocytes, resulting in CLD (40). Generally, TNF-α is a major factor contributing to the onset and prognosis of NAFLD because high TNF-α have been found in patients with NAFLD (41). Subsequently, in patients with ALD, the serum TNF-α levels were elevated, suggesting liver disease. The increased TNF-α levels in the diseased state of patients with CLD indicate its crucial role in the inflammation and pathogenesis of various CLDs.

We found that gut microbiota modulation therapies could reduce inflammation and immune response in patients with CLD. The TNF-α levels were significantly reduced in the interventional groups compared with the controls, with a significant effect occurring after treatment for 6 months, concluding that long-term therapy was beneficial compared with short-term. Similar to our study, a previous meta-analysis by Wang et al., which focused on probiotics in patients with NAFLD, TNF-α levels were significantly decreased in the interventional groups compared with the controls (42). The study also highlighted that the clinical benefits of gut-modulating therapies were more apparent with increased treatment time.

The clinical benefit of gut-modulating therapies in reducing pro-inflammatory cytokines was also apparent after analysis of CRP levels. We found that CRP levels were significantly reduced in patients receiving gut-modulating therapies compared with the controls. Similar results were reported by Pan et al., who determined that gut-modulating therapies reduced the inflammation in patients with NAFLD, specifically reducing CRP levels (43). Unlike other inflammatory cytokines, no significant difference was found in the IL-6 levels in both treatment groups. Furthermore, in some subgroup analyses, IL-6 significantly increased in the patients receiving gut-modulating therapies. Similar results were found by Kazimi et al., who established that IL-6 levels were significantly increased in patients receiving gut-modulating therapies (prebiotics and probiotics) compared with the controls (44).

Neutrophils are a critical component of the innate immune system. Over the years, it has been established that the interplay between gut microbiota and neutrophils interact to adjust the magnitude of neutrophil-mediated immunity (45). In liver disease, neutrophils are one of the significant innate immunity cells that have been associated with its pathogenesis (46). Empirical evidence from our included studies indicates that gut microbiota modulation may significantly reduce the pro-inflammatory state of the neutrophils. This, therefore, enables the neutrophils to have oxidative bursts upon stimulation (19). However, the effect on other immune cells, such as CD4+ and CD8+ lymphocytes, has yet to be observed.

## 5 Limitations

The objective of this review was to assess the efficacy of therapies targeting gut health in modulating the immune response in individuals with CLD. Pro-inflammatory markers are recognized as reliable indicators of inflammation and its variations; however, other measures, such as the activity of neutrophils and macrophages, can also effectively represent the immune response. Although certain studies reported changes in neutrophil activity, the available data were insufficient, precluding the aggregation of results (15) and limiting the ability to draw conclusions regarding the impact of gut microbiota-modulating therapies on cellular and innate immune responses.

## 6 Conclusion

This research demonstrates the potential of gut microbiota-targeted therapies in chronic liver disease (CLD) treatment. The interventions reduced pro-inflammatory cytokines, particularly TNF-α and CRP, suggesting alleviation of chronic inflammation in CLD. However, variability in IL-6 levels highlights the need for nuanced inflammatory marker monitoring. The findings have important clinical implications, opening possibilities for personalized CLD management strategies. Clinicians may consider these approaches as complementary or alternative treatments to enhance overall effectiveness. Future research should focus on the extended follow-up periods to observe long-term effects on gut microbiota composition and inflammatory markers. Comprehensive assessment of disease progression, including liver function tests and fibrosis markers. Developing personalized approaches based on individual patient characteristics while promising, these results underscore the need for continued investigation to fully exploit the benefits of gut microbiota modulation in managing the immune response in CLD.

## Data Availability

The original contributions presented in the study are included in the article/supplementary material. Further inquiries can be directed to the corresponding author.
